# Accurate image derived input function in [^18^F]SynVesT-1 mouse studies using isoflurane and ketamine/xylazine anesthesia

**DOI:** 10.1186/s40658-023-00599-8

**Published:** 2023-12-06

**Authors:** Alan Miranda, Daniele Bertoglio, Steven Staelens, Jeroen Verhaeghe

**Affiliations:** 1 Molecular Imaging Center Antwerp (MICA), University of Antwerp, Antwerp, Belgium; 2 Bio-Imaging Lab, University of Antwerp, Antwerp, Belgium

**Keywords:** Mouse, [^18^F]SynVesT-1, Image derived input function, Non-negative matrix factorization

## Abstract

**Background:**

Kinetic modeling in positron emission tomography (PET) requires measurement of the tracer plasma activity in the absence of a suitable reference region. To avoid invasive blood sampling, the use of an image derived input function has been proposed. However, an accurate delineation of the blood pool region in the PET image is necessary to obtain unbiased blood activity. Here, to perform brain kinetic modeling in [^18^F]SynVesT-1 dynamic scans, we make use of non-negative matrix factorization (NMF) to unmix the activity signal from the different tissues that can contribute to the heart region activity, and extract only the left ventricle activity in an unbiased way. This method was implemented in dynamic [^18^F]SynVesT-1 scans of mice anesthetized with either isoflurane or ketamine–xylazine, two anesthestics that we showed to affect differently radiotracer kinetics. The left ventricle activity (NMF-IDIF) and a manually delineated cardiac activity (IDIF) were compared with arterial blood samples (ABS), and for isoflurane anesthetized mice, arteriovenous (AV) shunt blood data were compared as well. Finally, brain regional 2 tissue compartment modeling was performed using IDIF and NMF-IDIF, and the model fit accuracy (weighted symmetrical mean absolute percentage error, wsMAPE) as well as the total volume of distribution (*V*_T_) were compared.

**Results:**

In isoflurane anesthetized mice, the difference between ABS and NMF-IDIF activity (+ 12.8 $\pm$ 11%, p = 0.0023) was smaller than with IDIF (+ 16.4 $\pm$ 9.8%, *p* = 0.0008). For ketamine–xylazine anesthetized mice the reduction in difference was larger (NMF-IDIF: 16.9 $\pm$ 10%, *p* = 0.0057, IDIF: 56.3 $\pm$ 14%, *p* < 0.0001). Correlation coefficient between isoflurane AV-shunt time activity curves and NMF-IDIF (0.97 $\pm$ 0.01) was higher than with IDIF (0.94 $\pm$ 0.03). The brain regional 2TCM wsMAPE was improved using NMF-IDIF compared with IDIF, in isoflurane (NMF-IDIF: 1.24 $\pm$ 0.24%, IDIF: 1.56 $\pm$ 0.30%) and ketamine–xylazine (NMF-IDIF: 1.40 $\pm$ 0.24, IDIF: 2.62 $\pm$ 0.27) anesthetized mice. Finally, brain *V*_T_ was significantly (*p* < 0.0001) higher using NMF-IDIF compared with IDIF, in isoflurane (3.97 $\pm$ 0.13% higher) and ketamine–xylazine (32.7 $\pm$ 2.4% higher) anesthetized mice.

**Conclusions:**

Image derived left ventricle blood activity calculated with NMF improves absolute activity quantification, and reduces the error in the kinetic modeling fit. These improvements are more pronounced in ketamine–xylazine than in isoflurane anesthetized mice.

**Supplementary Information:**

The online version contains supplementary material available at 10.1186/s40658-023-00599-8.

## Background

Kinetic modeling can be performed in positron emission tomography (PET) to quantify pharmacokinetic parameters of the PET tracers, such as total volume of distribution (*V*_T_) and binding potential [1]. In absence of a suitable reference region lacking specific binding to the target, kinetic modeling calculations require measurement of the amount of radiotracer in plasma over time, which serves as the input function to the kinetic model. This measurement can be performed by blood sampling at different time points during the scan, followed by further processing of the samples to extract the plasma tracer activity. Blood sampling is a challenging, invasive and time-consuming procedure, therefore other approaches have been devised to obtain the tracer plasma activity. One of such methods consists of measuring a blood pool region from the dynamic PET image, e.g., the heart or a large artery, to obtain an image derived input function (IDIF) [2].

In small animal PET studies, the IDIF has been also implemented to perform kinetic modeling [3, 4]. However, due to the small size of the animals, arteries are difficult to delineate. Usually the heart activity is used to measure the plasma activity, but delineation of the vena cava has also been performed to measure the IDIF in mice [5]. Within the heart, the left ventricle (LV) activity is used as the input function activity. Depending on the tracer uptake characteristics and preclinical scanner spatial resolution (e.g., volumetric spatial resolution $\sim$ 3.4 mm^3^ for the Inveon), tracer uptake in the surrounding heart regions, i.e., myocardium (MYO) and right ventricle (RV), can contaminate the left ventricle region (volume $\sim$ 5 mm^3^). This causes over/underestimation of the plasma activity, introducing bias in the kinetic microparameters [3–6]. Moreover, the small size of the heart in these animals hinder the delineation of the left ventricle of the heart, which is commonly performed manually [4, 5] or using radioactivity thresholds [3, 4, 6] with some guidance from an anatomical image (typically computed tomography, CT) if available. Additionally, even though the heart tissue can be located in a CT image, the contrast of the different heart regions is poor.

In order to separate the activity from the different heart regions in an unbiased way, source separation methods can be used, assuming the shape of the time activity curves of these regions is different. Factor analysis [7], independent component analysis [8] (ICA), non-negative matrix factorization [9] (NMF), and constrained NMF [10are some of the source separation methods than have been used in preclinical PET studies to segment the different regions of the heart. These methods have been used in tracers such as [^18^F]FDG [7, 9], [^13^N]ammonia [8], and [^11^C]acetate [7, 8].

In this work, we implemented a method to extract an IDIF that is closer to the ground-truth arterial input function for [^18^F]SynVesT-1, a tracer to image synaptic vesicle glycoprotein 2A as a proxy for synaptic density [4], in mice. Synaptic density PET tracers have been used to study multiple neurological disorders, presenting the possibility for new applications [11]. The volume of distribution of [^18^F]SynVesT-1 can be quantified using the 2 tissue compartment model (2TCM). Here, we compare an IDIF extracted by delineation of the mouse heart with an IDIF extracted using NMF source separation of the LV, MYO and RV activity. The IDIF activity is compared with blood samples activity and/or arteriovenous (AV) shunt whole blood activity. This comparison is performed in mice scanned either using isoflurane, or ketamine–xylazine anesthesia. Finally, 2TCM kinetic modeling *V*_T_ and goodness of fit are compared between fits performed using the heart delineation IDIF, and that obtained with NMF source separation.

## Methods

### PET scans

The experiments followed the European Ethics Committee recommendations (Decree 2010/63/CEE) and were approved by the Animal Experimental Ethical Committee of the University of Antwerp, Antwerp, Belgium (ECD 2020–59, 2020–71 and 2022–39). All scans were performed on Siemens Inveon PET/CT scanners (Siemens Medical Solutions, Inc., Knoxville, USA). For mice scanned under isoflurane anesthesia (3% for induction, 1.5% for maintenance, supplied with oxygen) 10 female C57BL/6J (group 1, 8 weeks old, body weight 18.8 $\pm$ 0.85 g) mice (Charles River, Lyon France) underwent a 2-h dynamic [^18^F]SynVesT-1 scan (injected dose 11.9 $\pm$ 2.5 MBq, injected mass 5.2 $\pm$ 0.8 nmol/kg) following i.v. tail vein tracer injection [12]. The bolus injection of the radiotracer was administered with an automated pump (Pump 11 Elite, Harvard Apparatus, USA) in a volume of 0.2 mL with a flow of 1 mL/min. A CT scan was performed after the PET scan for anatomical reference and to perform attenuation correction.

An additional group of 5 male C57BL/6J mice (group2, 10 months old, 34.4 $\pm$ 2.42 g; Jackson Laboratories) underwent surgery to insert an AV shunt into the femoral vein and artery to perform arterial blood sampling during the PET scan. Comparison of the plasma input function to the IDIF approach based on these data was previously published [4]. Briefly, after positioning the animal on the scanner bed under isoflurane anesthesia (3% for induction, 1.5% for maintenance), the AV shunt was connected to a peristaltic pump and to a coincidence detector (Twilite, Swisstrace) to measure whole blood activity with a 1 s resolution without blood loss due to a close loop to return blood back to the animal. Mice were administered with [^18^F]SynVesT-1 (13.4 $\pm$ 4.2 MBq, 4.22 $\pm$ 2.0 nmol/kg) and scanned for 2 h with simultaneous AV shunt blood sampling, followed by a CT scan. The bolus injection of the radiotracer was administered with an automated pump in a volume of 0.2 mL with a flow of 1 mL/min. The PET scan started 10 s before the radiotracer administration to capture the rise and peak of the input function. The activity measured with the detector was decay and background corrected, and cross-calibrated with the PET scanner each experimental day. To preserve the shape of the AV shunt TAC, noise reduction was performed using non-local means filtering. Since time of anesthesia with ketamine–xylazine is shorter (between 60 and 90 min) than the necessary time to perform the AV-sunt experiment (longer than 90 min), we did not perform the AV-sunt experiment in ketamine–xylazine anesthetized mice.

A group of 10 female C57BL/6J mice (group 3, Charles River, 8 weeks old, 18.4 $\pm$ 0.93 g) underwent dynamic PET scans using ketamine–xylazine (ketamine 150 mg/kg, xylazine 15 mg/kg, administered 30 min before tracer injection). Mice were administered with [^18^F]SynVesT-1 (9.15 $\pm$ 2.5 MBq, 6.4 $\pm$ 0.23 nmol/kg) and scanned for 1 h, following a CT scan. The bolus injection of the radiotracer was administered with an automated pump in a volume of 0.2 mL with a flow of 1 mL/min.

Dynamic PET scans were reconstructed with in-house developed OSEM list-mode reconstruction with 16 subsets and 16 iterations, considering spatially-variant resolution modeling [13]. Attenuation correction was performed using the µ-map calculated from the CT scan. Images were reconstructed using an image grid of 128 $\times$ 128 $\times$ 159 voxels (0.776 $\times$ 0.776 $\times$ 0.796 mm) in the $x$, $y$, and $z$ dimensions, respectively. Dynamic scans were reconstructed with a framing of 12 frames $\times$ 10 s, 3 $\times$ 20s, 3 $\times$ 30s, 3 $\times$ 60s, 3 $\times$ 150s, and 9 $\times$ 300s.

### Blood sampling and radiometabolites analysis

Blood extraction for whole blood activity measurement and radiometabolites analysis was performed in isoflurane and ketamine–xylazine anesthetized mice by cardiac puncture. The 20 mice from group 1 and 3 were used in this experiment, plus additional 4 mice (24 in total). All mice arrived at the laboratory facilities at the same time from the same vendor (Charles River) and had the same age (8 weeks old). The 24 mice were randomize in 2 groups of 12 mice: Twelve female mice (group 4, 21.8 $\pm$ 1.2 g) were administered with isoflurane (3% for induction, 1.5% for maintenance) 30 min before [^18^F]SynVesT-1 administration (7.32 $\pm$ 3.4 MBq) and blood was extracted using cardiac puncture at 2, 10, 30 and 60 min after tracer injection, using 3 mice per time point. The same procedure was performed in 12 female mice (group 5, 22.1 $\pm$ 0.96 g) administered with ketamine–xylazine (ketamine 150 mg/kg, xylazine 15 mg/kg) 30 min before [^18^F]SynVesT-1 administration (7.25 $\pm$ 3.2 MBq). Radiometabolites processing was performed as previously described [4, 12] to obtain the population-based plasma to whole blood ratio and the radiotracer parent fraction in plasma. For correction of all input functions, we interpolate to other time points performing a linear fit to the plasma to whole blood ratio and a sigmoid fit to the radiotracer parent fraction in plasma ratio [12]. For 10-month-old mice (group 2), we used the metabolites correction calculated for those mice in Bertoglio, et al. [4].

### IDIF extraction

The heart delineated IDIF was calculated as follows [3, 4, 6] in mice from group 1, 2 and 3. From the dynamic reconstruction aligned with the CT image, the heart was identified in the CT image and the PET frame with highest heart activity was determined. A spherical volume of interest (VOI) of 3.5 mm radius was drawn on the heart region and voxels within the VOI with an activity at least 50% the maximum activity within the VOI were considered to belong to the blood pool and were used to calculate the mean whole blood time activity curve (TAC).

### NMF for left ventricle activity extraction

Due to the positivity constraint of the PET data, we use non-negative matrix factorization (NMF) as the source separation method to identify the different heart regions (mice from groups 1, 2 and 3). However, since NMF converges to a local minimum, it is important to select a robust initialization method. Here we used initialization using non-negative ICA, since this method performs the best compared to a variety of other methods [14].

Initially, a matrix $Z$ is created by ordering in columns the individual voxel TACs considered in the spherical 3.5 mm VOI. Dimensionality reduction to the $n$ number of components that one wants to extract is performed by principal component analysis (PCA), i.e., using the matrix $B$ with the $n$ eigenvectors calculated from the covariance matrix of $Z$:

$$x={B}^{T}Z$$

After dimensionality reduction, whitening of the data is performed using the eigenvectors matrix $E$ and diagonal matrix $D$ from the covariance matrix of $x$ as described in Plumbley [15]:

$$z=E{D}^{-1/2}{E}^{T}x=Mx$$

The non-negative ICA algorithm [15] is used with data $z$ to obtain the demixing matrix $W$, and source matrix $y$, such that $y=Wz$. Mixing matrix $A$ in the original dimensions is calculated by undoing the whitening and dimension reduction operations:

$$A={{BM}^{-1}W}^{T}$$

Finally, ${G}_{0}=abs(A)$ and ${H}_{0}=abs(y)$ are the initialization matrices for the NMF [16]. The resulting matrices $G$ and $H$ ($Z\approx GH$) from the NMF contain the $n$ components TACs and corresponding weights, respectively, for every voxel considered in the heart VOI. The rows of matrix $H$ are reordered to obtain the probability images of every component.

### Identification of anatomical components

We assume 4 possible components can be included in the heart VOI voxels: left ventricle, right ventricle, myocardium, and contamination from the liver. Depending on the delineation of the VOI region, not all components might be included. For this reason, the NMF was calculated considering increasing number of components from 2 to 4 components. By visual inspection of the peak time and shape of the components TACs, as well as the shape of the probability images, it was determined which components were included in the VOI region. If increasing the number of components from $n$ to $n+1$ resulted in any 2 components TACs with the same peak time and/or spanning similar high probability voxels in the probability images, $n$ was considered as the correct number of identifiable components. The peak time of the right ventricle occurs before the peak time of the left ventricle, while peak time of myocardium occurs after the left ventricle peak time. The liver component TAC can easily be identified by its slow increase uptake.

To further assess if the obtained components are independent, the correlation of all components pairs TACs and probability maps can be performed. If the correlation between any 2 components is above a certain threshold, it can be considered that these 2 components are not independent, and therefore the number of components has to be reduced. In our case, we found empirically that a correlation coefficient threshold of 0.7 provides good results, although this threshold might need to be adjusted depending on the shape of the different components and probability maps.

The TACs of right ventricle, left ventricle, myocardium and/or liver were calculated from the average of the voxels selected from the probability images of the corresponding component. The relative probability ${r}_{ij}$ of each voxel $j$ belonging to component/region $i$ was calculated as:

$${r}_{ij}=\frac{{h}_{ij}}{\sum_{i}{h}_{ij}}$$

where ${h}_{ij}$ are the elements of matrix $H$ calculated with NMF. For region $i$, TACs from voxels with ${r}_{ij}$ > 0.9 were considered to calculate the average region $i$ TAC.

### Brain regional kinetic modeling

Previously, we have validated the 2-tissue compartment model (2TCM) for mouse [^18^F]SynVesT-1 brain kinetics [4]. In that study, 1TCM did not result in a good fit to the brain TACs. 2TCM was applied to calculate the total volume of distribution *V*_T_ in the brain regions cortex (CTX), caudate putamen (CP), thalamus (TH), hippocampus (HC), and cerebellum (CB) for mice in group 1, 2 and 3. The TACs of these regions were extracted as previously described [4, 12]. The input function used in the model fit was corrected for metabolites (either under isoflurane or ketamine–xylazine) and for plasma to whole blood ratio using population-based values calculated in the blood sampling and radiometabolites analysis experiment described above.

### Analysis

Sample size was defined in a previous study [12] where mean volume of distribution values with a standard deviation of 10% allowed used to detect significant relative differences of 10% with a statistical power of 0.8 between groups. No animals were excluded in the analysis. Normality of the samples was confirmed with a Wilk-Shapiro test.

The TAC extracted from the manual heart delineation (IDIF), and the left ventricle TAC calculated with NMF processing (NMF-IDIF,) for mice in groups 1, and 3, were compared with manual blood samples from mice in group 4 and 5, respectively, while IDIF and NMF-IDIF where compared with AV-shunt blood activity for mice in group 2. The difference of the standard uptake value (SUV) scaled blood samples at 2, 10, 30 and 60 min was calculated for IDIF and NMF-IDIF, for both isoflurane and ketamine–xylazine anesthetize mice in groups 4 and 5. For isoflurane AV shunt experiments (group 2), the difference between the AV shunt absolute activity, and the IDIF and NMF-IDIF activity, was calculated at the PET frames time points. Additionally, the shape similarity to the AV shunt curve was quantified by calculating the correlation between the AV shunt curve and IDIF and NMF-IDIF curves.

For the brain regional 2TCM kinetic modeling of isoflurane and ketamine–xylazine scans in groups 1 and 3, the goodness of fit was quantified either using the IDIF or the NMF-IDIF. A relative goodness of fit metric, i.e., the symmetric mean absolute percentage error (sMAPE), was used to be able to compare the fit in TACs with different levels of activity. In addition, the metric was weighted (wsMAPE) by the frame duration to consider the original, variable length, PET frames duration [17]:

$${\text{wsMAPE}} = \frac{{\mathop \sum \nolimits_{f} T_{f} \left| {{\text{TAC}}_{f} - C_{f} } \right|}}{{\mathop \sum \nolimits_{f} T_{f} \left| {{\text{TAC}}_{f} + C_{f} } \right|/2}}$$

where ${T}_{f}$ is the duration of PET frame $f$, ${\mathrm{TAC}}_{f}$ is the brain region TAC activity, and ${C}_{f}$ is the value of the curve fit to the 2TCM model. Finally, the wsMAPE and *V*_T_ calculated from the fit with IDIF or NMF-IDIF were compared using 2-way ANOVA analysis with correction for multiple comparison (Sidaks’s test) using GraphPad Prism 9.0. We report the *F* value (*F*_(degrees of freedom numerator),( degrees of freedom denominator)_) and the p value. Statistical significance was set at *p* < 0.05. GraphPad Prism 9.5 (GraphPad Software, California, USA) was used for the analysis (Table 1).

**Table 1:** Group assignment of the mice used in the different experiments

	Number	Sex	Age
Group 1: PET isoflurane scans	10*	Female	8 weeks
Group 2: PET isoflurane AV shunt	5	Male	10 months
Group 3: PET ketamine-xylazine	10*	Female	8 weeks
Group 4: Radiometabolites and blood sampling isoflurane	12	Female	8 weeks
Group 5: Radiometabolites and blood sampling ketamine-xylazine	12	Female	8 weeks

*These animals were randomized and also used for the radiometabolites and blood sampling experiments (group 4 and 5)

## Results

### Identification of anatomical components

**Fig. 1:**
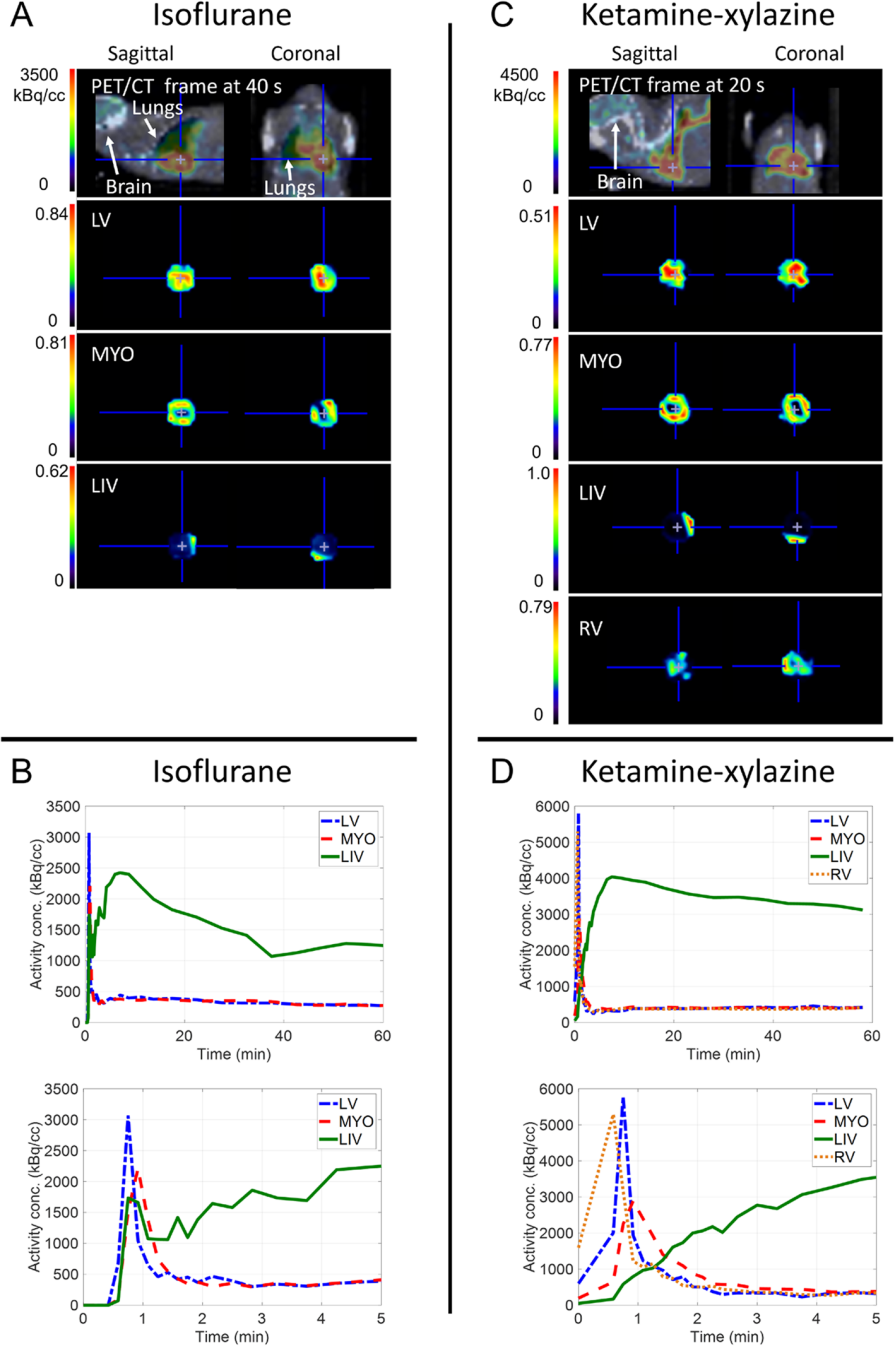
Example of a mouse frame (sagittal and horizontal planes) with highest heart activity (PET/CT top row), and the left ventricle (LV), myocardium (MYO), liver (LIV), and right ventricle (RV) probability maps calculated with the NMF processing, for a mouse under **A** isoflurane and **C** ketamine-xylazine anesthesia. The corresponding TACs associated with the different components are plotted, for **B** isoflurane and **D** ketamine-xylazine anesthetized mice, with a zoom at the first 5 min to show their peak activity

Figure 1 shows a representative mouse PET frame with highest heart activity, under isoflurane and ketamine–xylazine anesthesia, as well as the probability maps of the different components and corresponding TACs identified with the NMF processing. For both isoflurane and ketamine–xylazine anesthesia, the left ventricle (LV), and myocardium (MYO) components could be identified, and in some cases some contamination from the liver (LIV), or both liver and right ventricle (RV) for ketamine–xylazine, could be identified. The probability maps of the different components serve to identify the different regions, since they reflect the anatomical position of the corresponding regions. The components TACs shape provide additional information, which should correspond to the expected TAC shape of the corresponding anatomical region. The LIV contamination TAC could be easily identified due to its slow uptake, while the heart regions TACs were identified by their different peak times, with the RV peak time < LV peak time < MYO peak time.

### Comparison with whole blood activity

**Fig. 2:**
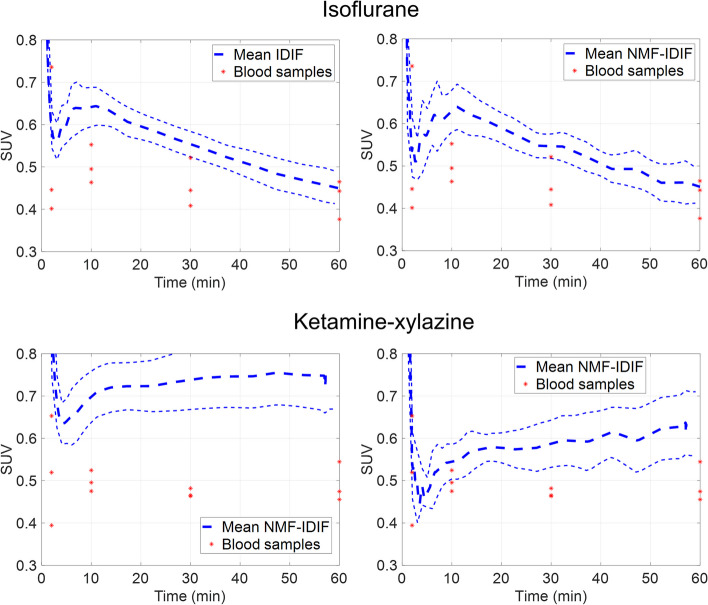
Mean IDIF TAC, and NMF-IDIF, together with heart whole blood samples, for isoflurane and ketamine-xylazine anesthetized mice. Light dashed lines indicate envelope of 1 SD

Figure 2 shows the mean IDIF and NMF-IDIF, together with the heart whole blood samples SUV. The difference between IDIF and the mean blood samples values at 2, 10, 30 and 60 min was 12.5%, 27.4%, 20.9%, and 4.96%, while for NMF-IDIF it was 1.5%, 25.0%, 19.4%, and 5.37%, respectively. For both IDIF and NMF-IDIF significant differences (*p* < 0.05) are found at 10 and 30 min, but not at 2 and 60 min. For ketamine–xylazine the difference between IDIF and NMF-IDIF was larger. At the same time points, the difference between the mean blood samples values and IDIF was 74.8%, 40.0%, 57.0%, and 53.5%, while for NMF-IDIF it was 5.87%, 9.32%, 24.9%, and 27.6%, respectively. For IDIF differences were significant (*p* < 0.05) at all time points while for NMF-IDIF only at 30 and 60 min.

**Fig. 3:**
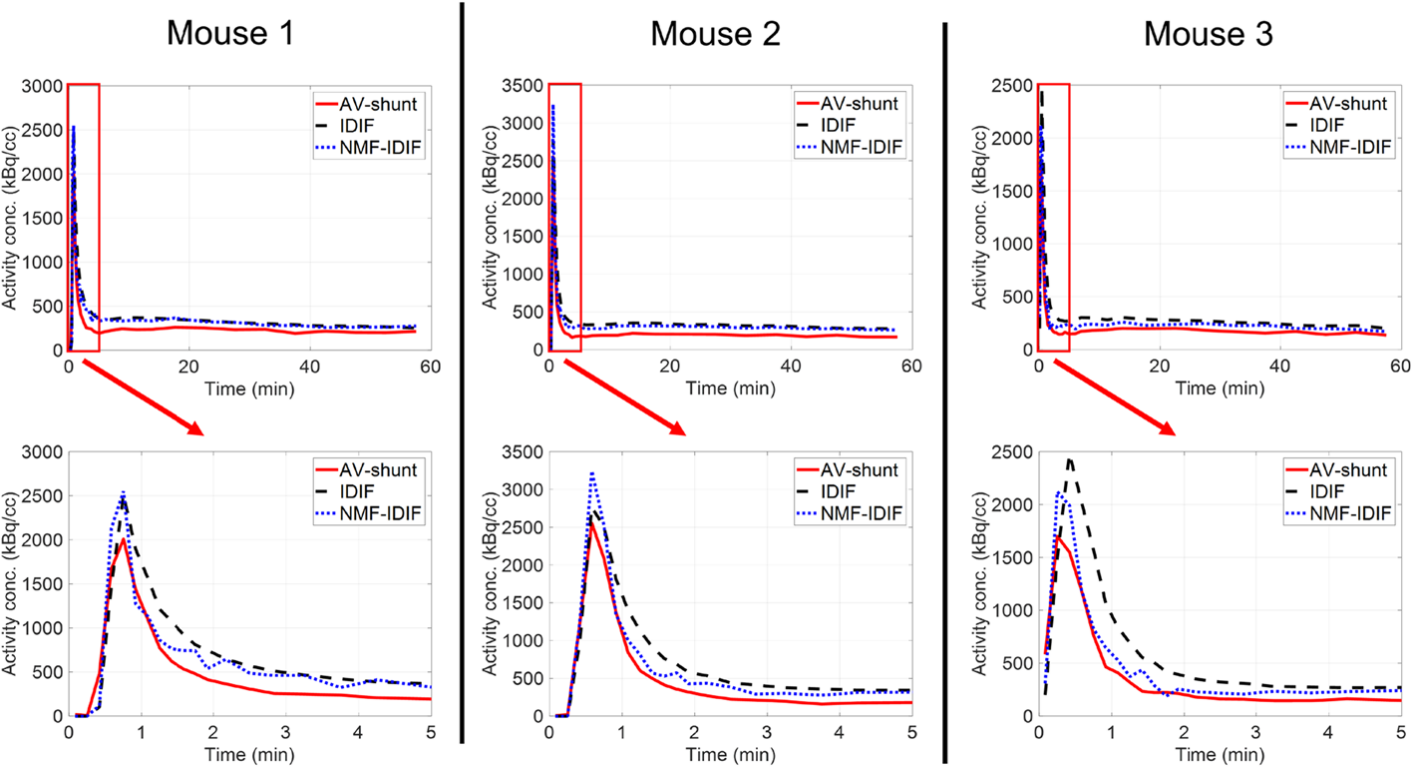
Comparison of isoflurane AV-shunt whole blood, IDIF and NMF-IDIF activity concentration for 3 different mice. Time zoom for the first 5 min shown in bottom row for visualization of peak and decay of the activity

Figure 3 shows the isoflurane AV-shunt, IDIF and NMF-IDIF whole blood activity for 3 out of 5 mice (mouse 4 and 5 in Additional file 1: Figure S6). Compared with IDIF, NMF-IDIF activity after the peak is slightly reduced, bringing it closer to the AV-shunt measured radioactivity. The activity wash-out after the peak shows better resemblance between NMF-IDIF and AV-shunt, while IDIF decay is slower. The integral of the measured activity is shown in Table 2 for the 3 different methods. Compared to the AV-shunt radioactivity measurement, the Bland–Altman analysis in Table 2 shows smaller bias and standard deviation for NMF-IDIF (+ 22.2 $\pm$ 3.28%) compared with IDIF (+ 35.7 $\pm$ 5.94%). Table 3 shows the correlation coefficient and mean relative difference, with respect to isoflurane AV-shunt activity, for IDIF and NMF-IDIF. In all mice, correlation was improved for NMF-IDIF compared to IDIF by 0.03 $\pm$ 0.02. Difference with AV-shunt radioactivity was reduced for NMF-IDIF compared with IDIF by 20 $\pm$ 14%.

**Table 2:** Integral of the SUV activity (0 – 60 min) for the different methods, as well as Bland–Altman analysis of IDIF and NMF-IDIF with respect to AV-shunt measured activity

	Activity integral (SUV∙min)	Bland-Altman bias $\pm$ SD (%)	95% limits of agreement
AV-shunt	29.6 $\pm$ 2.8		
IDIF	42.9 $\pm$ 3.1	+ 35.7 $\pm$ 5.94%	+ 47.4 – 24.1%
NMF-IDIF	37.5 $\pm$ 4.3	+ 22.2 $\pm$ 3.28%	+ 28.6 – 15.8%

**Table 3:** Correlation coefficient and relative difference between isoflurane AV-shunt activity concentration curve at every time point, vs IDIF and NMF-IDIF curves, for 5 different mice

	Mouse 1		Mouse 2		Mouse 3		Mouse 4		Mouse 5	
	Corr	Diff (%)	Corr	Diff (%)	Corr	Diff (%)	Corr	Diff (%)	Corr	Diff (%)
IDIF	0.949	58.4	0.952	42.8	0.972	73.6	0.900	74.7	0.938	70.7
NMF-IDIF	0.965	46.1	0.962	37.8	0.991	49.5	0.979	32.0	0.975	54.3

We additionally tested possible underestimation of the IDIF peak when 10 s frames are used in the initial time frames by comparison with quantification in shorter frames, i.e., 3 s in our test. Additional file 1: figure S1 shows the NMF-IDIF in the initial 2 min calculated in reconstructions with 10 s and 3 s frames. The peak was underestimated when 10 s frames were used. On average, an underestimation of 15.6% in the peak activity was found.

### AV-shunt brain regional kinetic modeling

@@@Additional file 1: figure S2 show the correlation plot of brain regional *V*_T_ values calculated using the AV-shunt input function vs IDIF, and AV-shunt vs NMF-IDIF. Compared with IDIF, NMF-IDIF improves person r correlation (IDIF: 0.908, NMF-IDIF: 0.914) and the linear fit slope is closer to the identity (IDIF: 0.68, NMF-IDIF: 0.80). The Bland–Altman plot of brain regional *V*_T_ values calculated with AV-shunt vs IDIF, and AV-shunt vs NMF-IDIF is shown in Additional file 1: figure S3. Using NMF-IDIF bias is reduced compared with IDIF (IDIF: -36.9%, NMF-IDIF: -29.0%), but standard deviation is larger (IDIF: 8.43%, NMF-IDIF: 9.92%).

### Isoflurane and ketamine–xylazine brain regional kinetic modeling

**Fig. 4:**
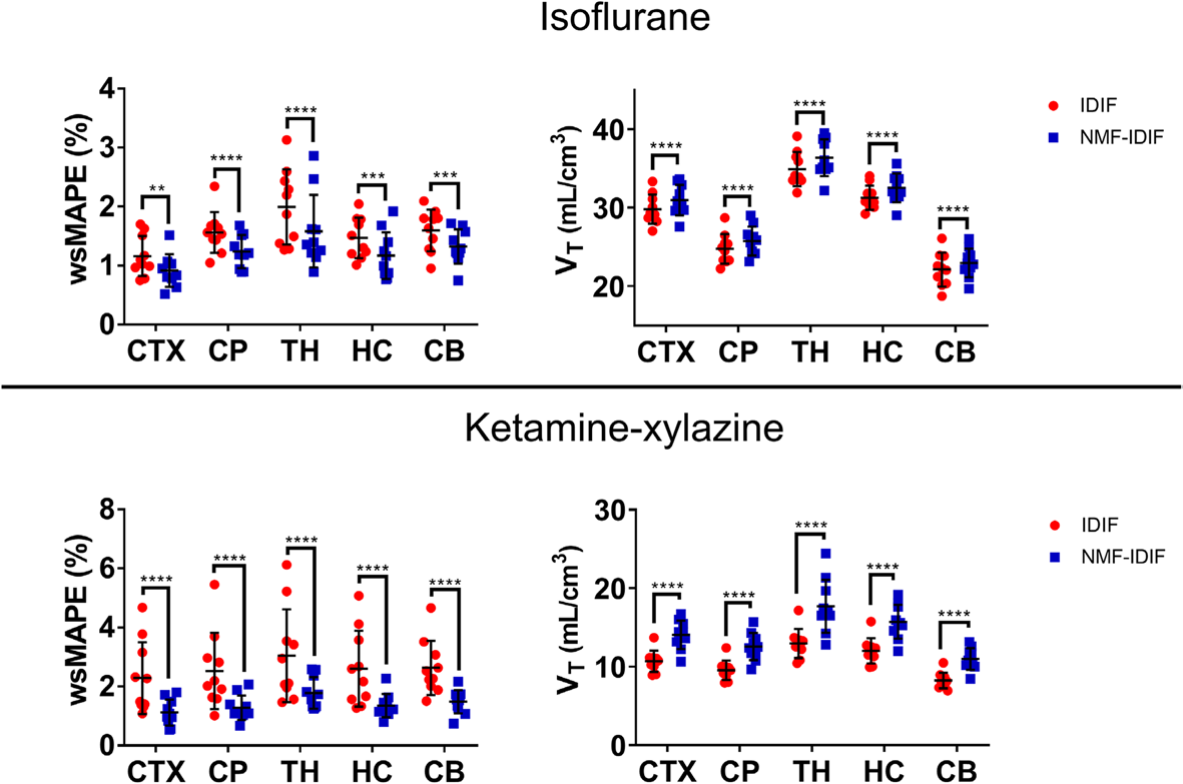
Weighted symmetrical mean absolute percentage error (wsMAPE) of the 2TCM fit, and total *V*_T_, for isoflurane and ketamine-xylazine anesthetized mice, using IDIF and NMF-IDIF as input functions. CTX: cortex, CP: caudate putamen, TH: thalamus, HC: hippocampus, CB: cerebellum. 2-way ANOVA analysis with correction for multiple comparison (Sidaks’s test). *N* = 10 in every group. p** < 0.01, p*** < 0.001, p**** < 0.0001

@@@@Additional file 1: figure S4 and S5 show the 2TCM fit in the 5 brain regions, for isoflurane and ketamine–xylazine anesthetised mice, using IDIF and NMF-IDIF in representative mice. Figure 4 shows the 2TCM wsMAPE using IDIF and NMF-IDIF, as well as *V*_T_, for mice under both anesthesia protocols for all brain regions. For isoflurane anesthetized mice there was a decrease in the 2TCM model fit error using NMF-IDIF as input function compared with IDIF. On average, there was a reduction of 0.31 $\pm$ 0.06% (F_1,9_ = 12.9, *p* = 0.0058) in wsMAPE using NMF-IDIF. A slightly, but significantly higher *V*_T_ was calculated using NMF-IDIF compared with IDIF (3.97 $\pm$ 0.13%, F_1,9_ = 10.9, *p* = 0.0093). For ketamine–xylazine anesthetized mice the decrease in 2TCM wsMAPE fit using NMF-IDIF compared with IDIF is more pronounced than in isoflurane anesthetized mice. wsMAPE was reduced 1.21 $\pm$ 0.05% (F_1,9_ = 10.4, *p* = 0.0104) using NMF-IDIF compared with IDIF. Additionally, for ketamine–xylazine the increase in *V*_T_ was larger than in isoflurane mice. Using NMF-IDIF, *V*_T_ was 32.7 $\pm$ 2.4% higher (F_1,9_ = 100.9, *p* < 0.0001) than when IDIF is used as input function (Table 4).

**Table 4:** Average total volume of distribution (*V*_T_), and 2TCM fit weighted symmetrical mean absolute percentage error (wsMAPE) for mice anesthetized with isoflurane or ketamine-xylazine

	Isoflurane				Ketamine-xylazine			
	*V*_T_ (ml/cm^3^)		wsMAPE (%)		*V*_T_ (ml/cm^3^)		wsMAPE (%)	
	IDIF	NMF-IDIF	IDIF	NMF-IDIF	IDIF	NMF-IDIF	IDIF	NMF-IDIF
CTX	29.8 $\pm$ 1.86	30.9 $\pm$ 1.94	1.16 $\pm$ 0.33	0.918 $\pm$ 0.27	10.6 $\pm$ 1.34	14.0 $\pm$ 1.81	2.28 $\pm$ 1.21	1.12 $\pm$ 0.44
CP	24.7 $\pm$ 1.90	25.7 $\pm$ 1.83	1.56 $\pm$ 0.34	1.23 $\pm$ 0.28	9.54 $\pm$ 1.25	12.5 $\pm$ 1.72	2.53 $\pm$ 1.28	1.28 $\pm$ 0.41
TH	34.9 $\pm$ 2.17	36.3 $\pm$ 2.35	1.99 $\pm$ 0.63	1.58 $\pm$ 0.61	12.9 $\pm$ 1.84	17.6 $\pm$ 3.37	3.04 $\pm$ 1.57	1.78 $\pm$ 0.53
HC	31.2 $\pm$ 1.54	32.5 $\pm$ 1.83	1.47 $\pm$ 0.34	1.17 $\pm$ 0.39	12.0 $\pm$ 1.62	15.7 $\pm$ 2.15	2.60 $\pm$ 1.28	1.35 $\pm$ 0.39
CB	22.1 $\pm$ 2.15	22.9 $\pm$ 1.83	1.59 $\pm$ 0.35	1.32 $\pm$ 0.29	8.23 $\pm$ 0.99	10.9 $\pm$ 1.39	2.63 $\pm$ 0.91	1.48 $\pm$ 0.39

*CTX* cortex, *CP* caudate putamen, *TH* thalamus, *HC* hippocampus, *CB* cerebellum

## Discussion

Kinetic modeling using dynamic PET data requires knowledge of the tracer plasma activity over time to perform the fit to compartmental models. However, measurement of the plasma tracer activity through blood sampling is challenging, especially in small animals in which the blood volume extraction is limited to avoid harm to the animal. For this reason, using an image derived input function greatly improves practicality in PET kinetic modeling. Here, we developed a method to improve the quantification of the IDIF for [^18^F]SynVesT-1 in animals under isoflurane and ketamine–xylazine anesthetics by using signal unmixing methods to differentiate the different tissue activities that can contribute to the IDIF. An accurate quantification of plasma activity is necessary to obtain kinetic model parameters without bias. The NMF-IDIF shows better correspondence with blood samples radioactivity, and reduced the error in the kinetic modeling fit, compared with IDIF.

The use of NMF with non-negative ICA initialization to unmix the different tissue activities that could be included in the heart IDIF produced components which corresponded well with the expected anatomical regions when used with [^18^F]SynVesT-1 dynamic PET data. Both the voxels spatial location (confirmed with CT image), and time activity curves shape, corresponded well with the expected results. When 3 different components TACs could be identified in the heart voxels, the TACs with the first, second and third earliest peaks corresponded spatially with the expected right ventricle, left ventricle, and myocardium shapes and location [9], respectively. If a component TAC with slow uptake could be identified, the voxels spatial location of this component was in the border between the heart and the liver, indicating radioactivity contamination from the liver to the heart activity.

To automate the process to select the optimal number of components in NMF we proposed a simple approach consisting on inspection of the the correlation between the different components and defining a threshold above which we can assume the components are no longer independent. Since the threshold has to be defined empirically, this has to be done in a case by case basis limiting its application. There are many other, more computationally expensive, methods reported in the literature to select the optimal number of components in NMF, such as cross validation [18]. For the purpose of this study visual inspection of the components TACs and probability maps was sufficient to accurately identified the correct number of components.

Kinetic modeling 2TCM *V*_T_ calculated with the AV-shunt input function had better correspondence with values calculated using NMF-IDIF compared with IDIF. Correlation was improved and Bland–Altman bias was reduced. However, some bias remains using NMF-IDIF, which could be caused by the effects described which cause positive bias in the input function activity.

In theory, the left ventricle could be manually delineated, but due to the short duration of the initial PET time frames in which the heart activity is visible, the noise in the image hinders this delineation. For later frames (approximately 2 min after injection time) in [^18^F]SynVesT-1 images, the heart activity is no longer visible. Moreover, variability can increase due to differences in manual delineation. The automated processing proposed here allows for a more reproducible and accurate left ventricle definition compared to manual delineation.

The TACs of the different heart regions were calculated as the average of their voxels activities, with the different heart regions defined using the NMF probability maps information. Although matrix $G$ calculated with NMF contains the respective components TACs, including left ventricle, we observed that these TACs could present a shape that slightly deviated from the respective voxels tissue TACs. This could have occurred due to noise, and suppositions in the independent components calculation (e.g., orthogonality of the independent components) which might not be exactly fulfilled in real-world data. Therefore the average voxels TACs were used to preserve the shape and activity scale.

For ketamine–xylazine anesthetized mice the right ventricle, left ventricle, and myocardium tissues could be identified, while for isoflurane anesthetized mice only left ventricle and myocardium tissues were identifiable. It has been previously reported that ketamine–xylazine reduced the heart rate of mice to almost half the heart rate in mice anesthetized under isoflurane anesthesia [19, 20], as well as reduced heart ejection fraction [21] under ketamine–xylazine. These physiological changes under ketamine–xylazine might slow down the heart blood exchange kinetics, causing the peak uptake time of the different heart tissues to have enough separation in time to be identifiable in the PET sampling. On the other hand, the faster heart kinetics under isoflurane anesthesia do not allow to differentiate the right and left ventricle TACs in the PET sampling time. For example, for [^18^F]FDG scans of mice anesthetized with isoflurane anesthesia, the difference between the peak activity time of RV and LV is about 1 second [22]. In all cases when liver contamination was present, the liver TAC was easily identified due to its slow uptake compared with the heart uptake.

The difference between the blood samples radioactivity (taken at 2, 10, 30 and 60 min) and the left ventricle IDIF (NMF-IDIF) was smaller than between the raw IDIF (i.e., without tissue differentiation). For isoflurane anesthetized mice this difference was slightly reduced by using NMF-IDIF, but for ketamine–xylazine anesthetized mice the difference was greatly reduced. The error between blood samples and the IDIF activity was particularly improved at the portion of the IDIF after the peak, corresponding to blood samples at 2 min, when the NMF-IDIF was compared in both anesthetics. This is due to the exclusion of myocardium activity in the NMF-IDIF, which cause a slower IDIF activity wash-out. This effect is clearly observed in the comparison with the AV-shunt whole blood activity in which the peak and decay portion of the TAC overlap better for NMF-IDIF in comparison with IDIF.

The whole blood TAC late phase activity was also reduced in NMF-IDIF compared with IDIF, with larger reduction in ketamine–xylazine compared with isoflurane anesthetized mice. This indicates that LV surrounding tissue (i.e., right ventricle and myocardium) activity concentration is larger when ketamine–xylazine is used in comparison with isoflurane. Looking at the blood samples and AV-shunt whole blood radioactivity, however, some positive bias is observed in the NMF-IDIF late phase under isoflurane anesthesia. Since the mouse LV region is small compared with the scanner spatial resolution, the partial volume effect could introduce some residual bias in this region. Instrumental calibration uncertainties of the gamma counter and PET scanner could have also contributed to these differences. In addition, dispersion in the AV-shunt line could have caused underestimation of the whole blood activity [23]. Although the NMF-IDIF peak was underestimated in 10 s frames compared with 3s, the difference was not large, and should not impact kinetic modeling, as observed in the 2TCM model fit.

Although noise in the NMF-IDIF was larger than in the manually delineated IDIF, which is calculated considering the average of more voxels, the kinetic modeling fit did not seem to be impacted. On the contrary, the error in the modeling fit was improved when the NMF-IDIF was used. The inclusion of myocardium activity in IDIF, which presents later peak time and slower activity decay, changed the input function shape, which in turn affected the fit of the 2TCM to brain regional activity. This is observed in the larger fit error when IDIF was used compared with NMF-IDIF (only LV activity). For ketamine–xylazine anesthetized mice this improvement in the 2TCM model fit was more pronounced than in isoflurane anesthetized mice. The slower heart kinetics under ketamine–xylazine cause the inclusion of surrounding tissue activities to deform to a higher degree the shape of the left ventricle TAC, causing larger fit error when using the manually delineated IDIF.

For isoflurane anesthetized mice, the *V*_T_ in the different brain regions was slightly increased ($+$ 3.99%, *p* < 0.0001) using NMF-IDIF in comparison with *V*_T_ calculated with IDIF. On the other hand, for ketamine–xylazine anesthetized mice the increase in *V*_T_ when NMF-IDIF is used was higher ($+$ 30%, *p* < 0.0001) in comparison with* V*_T_ calculated with IDIF. This increase in *V*_T_ is expected since the plasma activity integral is reduced when NMF-IDIF is considered in comparison with IDIF.

Overall, kinetic modeling using the left ventricle activity (i.e., NMF-IDIF) produced improved 2TCM parameters compared with a manually delineated IDIF in isoflurane anesthetized mice. Similarly, for ketamine–xylazine anesthetized mice is recommended to use an accurately delineated left ventricle activity for the kinetic modeling since the heart kinetics are greatly affected (slower heart and reduced ejection fraction) by this anesthetic [19–21].

### Limitations

In this implementation of the NMF, we defined the number of optimal components by visual inspection. However, automated algorithms can be implemented to define the optimal number of components [18]. In addition, due to the limited time of anesthesia of ketamine–xylazine (60–90 min), we did not perform the AV-shunt experiment with this anesthetic. The AV-shunt data was not corrected for dispersion, so the peak activity could have been underestimated, as previously described [23]. Finally, improvement using NMF-IDIF compared with IDIF is larger in ketamine–xylazine anesthetized mice compared with isoflurane anesthesia.

## Conclusions

We implemented a method using non-negative matrix factorization to extract the heart left ventricle activity from [^18^F]SynVesT-1 PET dynamic images, in mice anesthetized with isoflurane and ketamine–xylazine anesthesia. Compared with arterial blood samples, the left ventricle whole blood activity calculated with the proposed method has better similarity than a manually delineated image derived input function. This method allows the non-invasive extraction of the input function with reduced bias, improving kinetic modeling quantification and practicality in longitudinal studies performed with [^18^F]SynVesT-1.

### Supplementary Information

**Additional file 1.** Supplementary figures.

## Data Availability

The datasets used and/or analyzed during the current study are available from the corresponding author on reasonable request.

## Abbreviations

PET: Positron emission tomography
*V*_T_: Total volume of distribution
IDIF: Image derived input function
LV: Left ventricle
MYO: Myocardium
RV: Right ventricle
CT: Computed tomography
ICA: Independent component analysis
NMF: Non-negative matrix factorization
PCA: Principal component analysis
2TCM: 2 Tissue compartment model
AV: Arteriovenous
TAC: Time activity curve
VOI: Volume of interest
CTX: Cortex
CP: Caudate putamen
TH: Thalamus
HC: Hippocampus
CB: Cerebellum
wsMAPE: Weighted symmetric mean absolute percentage error
