# Supplementary material for: Accurate image derived input function in [18F]SynVesT-1 mouse studies using isoflurane and ketamine/xylazine anesthesia
Source: EJNMMI Phys. 2023 Dec 6;10:78. doi: 10.1186/s40658-023-00599-8 (PMC10697927; doi:10.1186/s40658-023-00599-8)
Supplement: Supplementary file 1 — Additional file 1. Supplementary figures. [file 40658_2023_599_MOESM1_ESM.pdf]

Accurate image derived input function in [^{18}F]SynVesT-1 mouse studies using isoflurane and ketamine/xylazine anesthesia

Alan Miranda[1*], Daniele Bertoglio[1,2], Steven Staelens[1], Jeroen Verhaeghe[1]

[1]*Molecular Imaging Center Antwerp (MICA), University of Antwerp, Belgium*

[2]*Bio-Imaging Lab, University of Antwerp, Belgium*

**Corresponding author: alan.mirandamenchaca@uantwerpen.be*

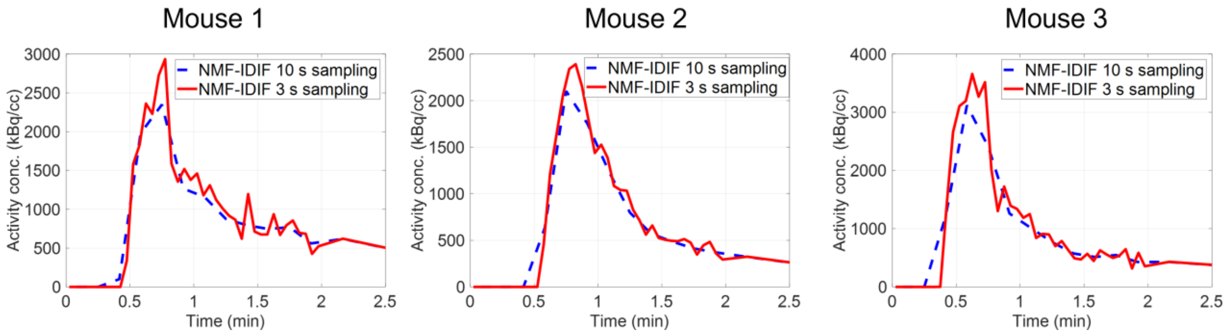

Supplemental figure 1. Comparison of NMF-IDIF calculated with time frames of 10 s in the first 2 minutes of the scan, with NMF-IDIF calculated with time frames of 3 s in the first 2 minutes.

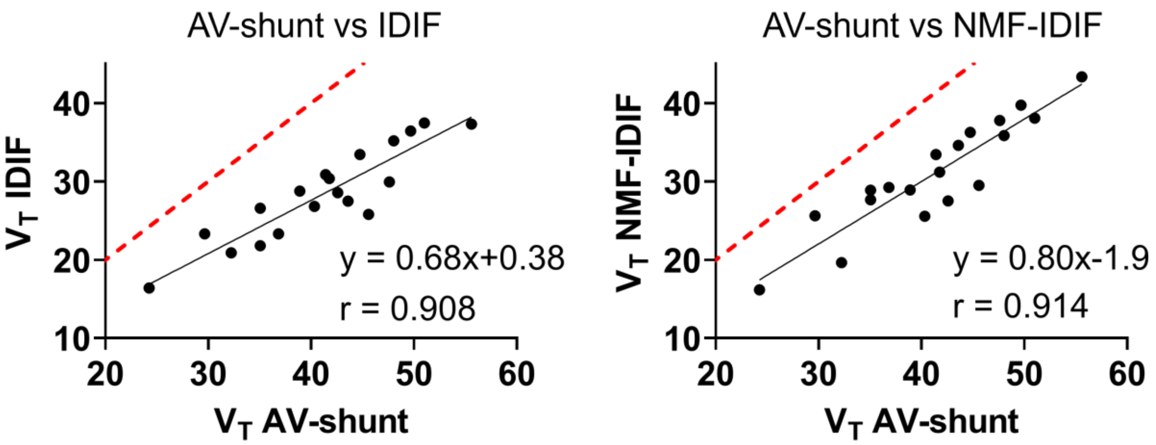

Supplemental figure 2. Correlation and linear fit of brain regional 2TCM V_T values calculate using the AV-shunt input function vs IDIF input function, and AV-shunt input function vs NMF-IDIF input function. Red dashed line indicates the identity line.

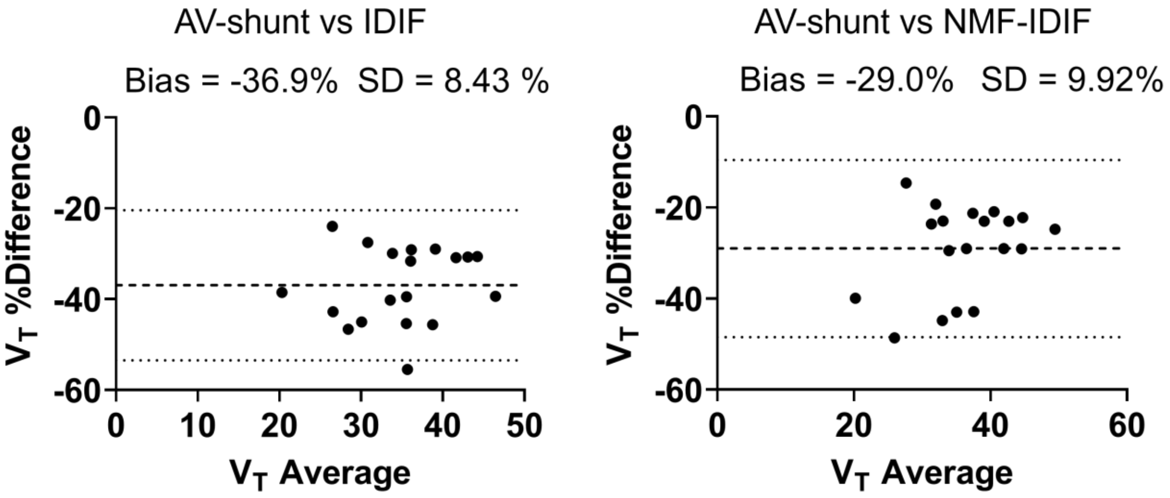

Supplemental figure 3. Bland-Altman plot of 2TCM V_T values calculate using the AV-shunt input function vs IDIF input function, and AV-shunt input function vs NMF-IDIF input function. Dotted lines indicate 95% confidence intervals and dashed line indicated the bias.

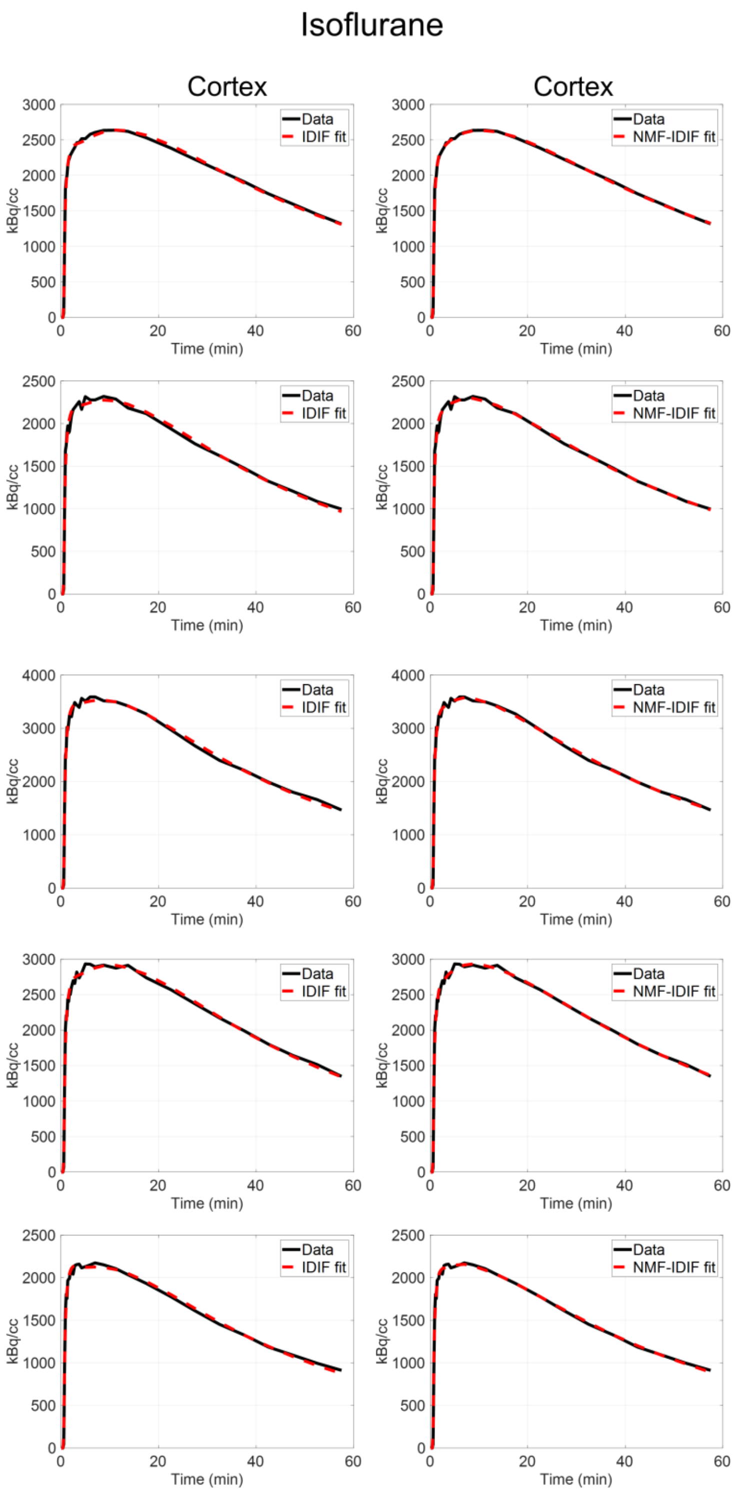

Supplemental figure 4. Brain regional time activity curves and 2TCM fit using the IDIF and the NMF-IDIF for a representative mouse under isoflurane anesthesia.

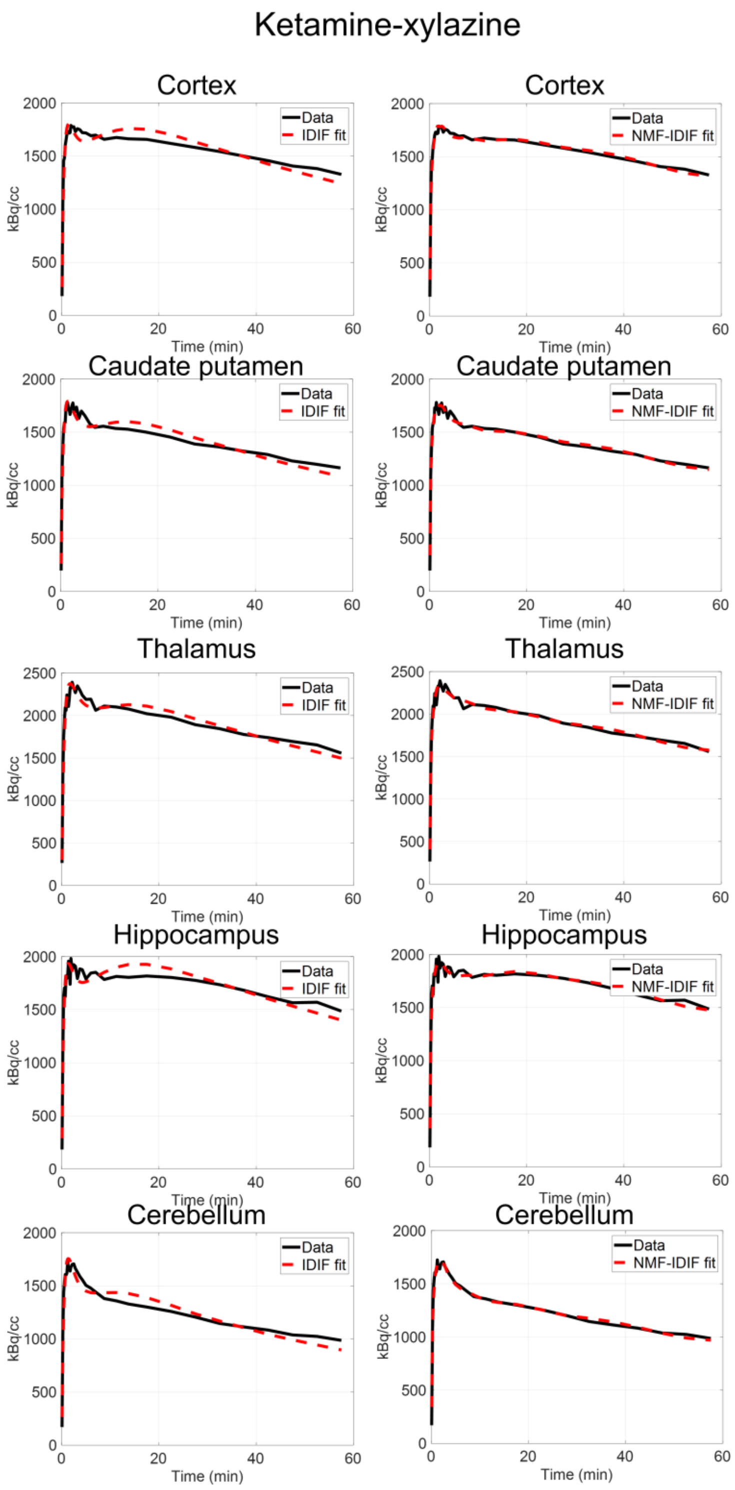

Supplemental figure 5. Brain regional time activity curves and 2TCM fit using the IDIF and the NMF-IDIF for a representative mouse under ketamine-xylazine anesthesia.

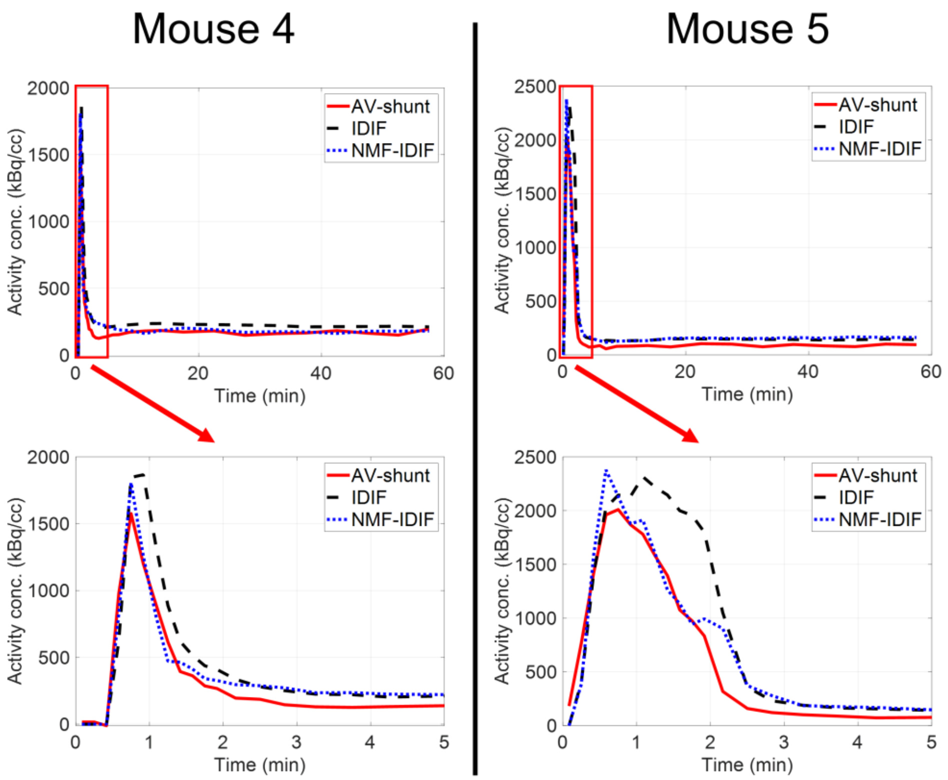

Supplemental figure 6. Comparison of isoflurane AV-shunt whole blood, IDIF and NMF-IDIF activity concentration for 2 different mice. Time zoom for the first 5 minutes shown in bottom row for visualization of peak and decay of the activity.
